# Plastics and Microplastic in the Cosmetic Industry: Aggregating Sustainable Actions Aimed at Alignment and Interaction with UN Sustainable Development Goals

**DOI:** 10.3390/polym14214576

**Published:** 2022-10-28

**Authors:** Anelise Leal Vieira Cubas, Ritanara Tayane Bianchet, Izamara Mariana Aparecida Souza dos Reis, Isabel C. Gouveia

**Affiliations:** 1 Environmental Science Master’s Program, University of Southern Santa Catarina (Unisul), Avenida Pedra Branca, 25, Palhoça 80137270, Brazil; 2 Environmental Engineering, University of Southern Santa Catarina (Unisul), Avenida Pedra Branca, 25, Palhoça 80137270, Brazil; 3 FibEnTech R & D—Fiber Materials and Environmental Technologies, Universidade da Beira Interior, Rua Marquês d’Avila e Bolama, 6201-001 Covilhã, Portugal

**Keywords:** cosmetics industry, plastics and microplastic, sustainable development goals

## Abstract

Excessive use of petroleum derivatives in cosmetics, whether in compositions or packaging, predominating the use of plastics, parabens, microplastics and other polymers, has had negative environmental impacts. The cosmetics market has gained prominence in recent years and bioeconomy and circular economy policies are putting pressure on the market to use bio-based and biodegradable materials. In this context, the objective of this review article is to provide an overview of how the aggregation of sustainable actions in the cosmetic industry contributes to the fulfillment of the 2030 Agenda and how this can serve as a guide in building a more resilient and sustainable society. For that, the generation of residues during the production processes was examined and the environmental problems generated by the cosmetic industry were addressed. Then, the role of aggregating sustainable actions and innovations with regard to the achievement of the UN Sustainable Development Goals (SDGs) in the cosmetic industry were evaluated.

## 1. Introduction

The global cosmetics market lies in approximately third place in terms of fastest growth, and the market for ecologically-based cosmetics is estimated to reach US $5.25 billion by 2029, based on a growth forecast with a compound annual rate of 5.18% during the forecast period of 2019 to 2029 [1]. The personal care and beauty sector is characterized by maintaining relations with several other fields of production (chemical, food and pharmaceutical), all responsible for product development. The area of materials development, such as packaging, is also strongly linked since it forms part of the design of cosmetics. Packaging can be comprised of a great diversity of materials, including cardboard and glass, but it is predominantly made from plastic. The packaging guarantees the brand identity and is widely used as a marketing strategy in the cosmetics industry [2].

Regarding consumers, women account for approximately 79% of cosmetic product use, for beauty, hygiene and care purposes, and moisturizers, deodorants and hair and makeup products are the most notable [2].

In view of the socioeconomic model that prevails worldwide, capitalism together with globalization has promoted increased consumption, associating happiness with a volume of material acquisitions. Consumer demand is driving the need to replace consumer goods and raw materials with more ecologically friendly options, since many components are extracted directly from nature, which has its own cycles. In parallel with consumption, there is rapid disposal, which is not controlled since it is carried out by the consumer, and the waste may end up in landfills or in inappropriate places. Therefore, it is clear that the greatest impacts of consumerism are the uncontrolled increase in the use of natural resources and the generation of waste [3,4].

With the increase in consumerism, the cosmetic industry has not been left behind, gaining an increasing number of consumers and a high level of demand, but this comes with socio-environmental consequences. Currently, the generation of plastics and microplastics (MPs) is one of the most serious environmental issues, particularly that associated with the cosmetic industry. These plastics can be present in a primary form, that is, as components of the formulations, such as exfoliating agents, or as secondary plastics derived from cosmetic packaging. Microplastics are particles of approximately 5 mm to 0.1 μm, which are difficult to filter out in wastewater treatment plants, reaching water courses and draining into the oceans. These microparticles are easily consumed by marine animals, plankton and other biota, negatively impacting the entire food chain of the marine ecosystem [5,6].

Plastics have some characteristics that, from an environmental point of view, are not attractive, such as high resistance to biodegradation and good adsorption properties [6]. This causes great concern to researchers since these PMs can adsorb persistent organic pollutants (POPs) through accumulation, and their low biodegradability increases the risk of an almost irreversible imbalance in the marine system, especially when these particles are ingested by plankton, which are the primary consumers in the marine food chain [6,7].

In addition to plastics and microplastics, another issue to be considered is how to treat the residues generated in the production processes of the cosmetics industry. Petroleum derivatives, heavy metals, dyes, surfactants, detergents and other components need to be treated and handled responsibly to avoid ecological changes resulting from the disposal of wastewater or other types of waste.

As an effective alternative to building a more environmentally responsible, socially equitable and economically accessible industry, the Sustainable Development Goals were established. These represent a global landmark, involving the coming together of countries that make up the United Nations (UN) to create objectives and goals that guarantee more sustainable development, allowing future generations to enjoy the same quality of life as we do today. The summit meeting was held in New York from 25 to 27 September, 2015. The objectives formed part of a new action agenda planned for 2030, based on the progress and lessons learned from the Millennium Development Goals (MDGs), between 2000 and 2015. The 2030 agenda is the result of joint efforts by several countries to seek the building of a more egalitarian, fair future, attentive to climate change, ready to mitigate harmful impacts on the environment, with a view to cleaner energies, promoting well-being and prosperity for all [8,9].

In this context, the purpose of this review paper is to provide an overview of how aggregating actions in the cosmetics industry could contribute to the achievement of sustainable development objectives and how they can act as a guide in the building of a more sustainable industry. The issues are addressed in the following sections: Section 2 describes the production of cosmetics on an industrial scale and the generation of waste from this process; Section 3 deals with the environmental problems generated by the cosmetics industry; Section 4 reports the aggregation of sustainable actions and innovations in the cosmetic industry; Section 5 addresses how the Sustainable Development Goals (SDGs) provide direction for the cosmetics industry; and the final considerations of the study are then presented.

## 2. Production of Cosmetics on an Industrial Scale

According to the European Commission, a product can be considered a cosmetic when there is “any substance or mixture intended for use on external parts of the body, such as nails, lips, epidermis, hair and external parts of the genitals, or teeth and mucous membranes, with the exclusive objective of cleaning, perfuming, protecting, altering appearance, and maintenance or correction of body odors ” [10].

**Figure 1:**
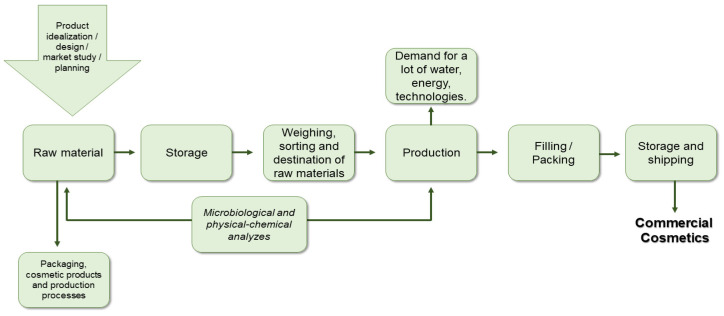
Stages involved in the cosmetics production process. Source: Authors, adapted from PEREIRA, F. S. G. (2010) [12].

According to data published in the ABIHPEC 2021 yearbook, Brazil has 3168 cosmetics industries registered with the national health surveillance agency. Globally, this number could be ten times higher, demonstrating how the demand for cosmetics reaches a large consumer audience. In view of this, it is of interest to describe the production stages of these highly popular products [11]. Figure 1 provides general details of this process.

Firstly, the product is idealized, researched and a plan for production is obtained. The raw materials required for the cosmetic itself and its packaging are then selected, extracted and stored. These materials are then weighed and they are destined for their production end. Both the initial raw materials and the final product undergo physical-chemical analysis to record possible organoleptic changes, and microbiological tests are conducted to ensure that the cosmetics are not contaminated by pathogens. Finally, the products are appropriately packaged and sent for storage and shipping [12]. Samples are always taken from each batch produced to guarantee the quality of the remaining cosmetics distributed for trade.

During the production process, as in other industries, a significant amount of water is required, since it is a constituent part of cosmetics, predominantly used as a component solvent. In addition, it is widely used in the cleaning of equipment, pipes and floors and for cooling [13]. Consequently, it becomes contaminated during use, both with dirt from machinery, as well as by the additive products for hygiene, such as bleach. Therefore, wastewater is characterized as the industrial liquid effluent with greatest predominance in the cosmetic industry [14]

The energy demand of the cosmetic industry is usually low, mainly because most of the processes are carried out at room temperature, due to the characteristics of the product, as they contain delicate raw materials that are easily degraded, for example, at temperatures higher than 80 °C [12]. The processes expected to need the most energy are the extraction of raw materials and distribution of the final product, and these require the use of petrochemical derivatives.

## 3. Environmental Problems Associated with the Cosmetics Industry

### 3.1. Wastes from Cosmetics Production Processes

The expansion of the cosmetics industry has led to the generation of a greater amount of waste in all stages of the production and disposal of cosmetics. The workers at production plants must remain vigilant to avoid the unintentional discharge of its effluents since they may be loaded with various chemical agents. An example is surfactants, which act as an amphiphilic medium, allowing the mixture of water and oil. These agents, when disposed of incorrectly in wastewater cause numerous harmful effects, notably the formation of bubbles that prevent gas exchange, directly affecting the photosynthesis of aquatic organisms [15].

Consumers value quality products that are long lasting. Thus, cosmetics contain preservatives and antioxidants in their composition, to prevent the proliferation of harmful micro-organisms and oxidation of products. In this industry, the use of parabens derived from oil as a preservative has become the subject of discussions on how this component can affect our health, in the short and long term. The environmental impact of parabens and other derivatives of oil is also an issue of concern. This discussion encompasses the viewpoint of the consumers, who are increasingly more aware of environmental, welfare and self-care guidelines [16]

As mentioned above, the use of parabens in cosmetology is abundant, and we are exposed to these compounds through the application of products to the skin, where these particles penetrate regardless of their solubility. In these circumstances, cosmetovigilance emerges as a form of public health surveillance for monitoring and detecting possible side effects associated with the use of cosmetics. Toxicological tests to determine carcinogenicity and phototoxicity, among other possible effects, are used to assess which compounds are safe for cosmetic application [17].

With regard to parabens and their influence on health, studies highlight concerns about the endocrine system, given their classification as hormonal disruptors, with affinity for estrogen, androgen and even thyroid receptors. In addition, mitochondrial dysfunction resulting from oxidative stress could interfere with homeostasis of the body [16]. Dyes, such as coal tar, are other petroleum derivatives, which consist of a combination of aromatic hydrocarbons, nitrogenous bases and phenols, plus a color and a number, such as tartrazine, quinoline yellow and alizarin green, and reports have indicated reactions leading to dermatitis and cancer [18].

Another cosmetic component with a high environmental impact is titanium dioxide (TiO_2_), present mainly in sunscreen. When discarded in water bodies it absorbs and reflects sunlight that enters the environment, affecting the growth and survival of aquatic animals and interfering in the photosynthesis of the ecosystem flora, including in coral reefs. Coupled with this component, there is the commonly used chelating agent EDTA, which has high toxicity. Studies have demonstrated its ability to inhibit cell division, chlorophyll synthesis and algal biomass production [19].

As with all residual waters, that of the cosmetic industry shows variations in the hydrogen potential (pH). When it is highly alkaline it becomes harmful in water resources, and a corrective solution is needed to reduce the pH to tolerable levels, with the use of inorganic, sulfuric and hydrochloric acids. At a pH below 5 and above 9, cellular activity in living beings tends to be reduced and some cannot survive under these conditions [20]. Thus, it is extremely important to adjust the pH to an appropriate level.

All waste generated in the production process must be properly treated as the effluents in this industry also have high chemical oxygen demand (COD) and contain organic compounds with low biodegradability. Initially, physical-chemical treatment is carried out to remove the organic compounds and inorganic material, with the addition of coagulants/flocculants and pH correctors. This is followed by the separation of the flakes by decantation, and the treatment ends with filtration. Tones (2020) reports other effluent treatment methods adopted, such as the application of advanced oxidative processes (POA), ultrafiltration, anaerobic upflow reactors, coagulation-Fenton, photo-Fenton, EC—TiO_2_ and membranes, but notes that the results of studies are for batch tests and not continuous flow systems [21]. Therefore, the ideal treatment is directed according to the availability of space, where the company is located and its surroundings and, most importantly, the cost of the implementation of these systems [22].

Research is being carried out to add value to these cosmetic industrial residues. Purwanto and Permana-Citra (2019), for example, prepared paving blocks using residual paints/dyes as raw materials. The strength of the blocks was tested and it was demonstrated that 5% of residue provides increased resistance (up to 10 MPa), according to the pattern of use as paving for pedestrians (at the same time reducing the cost of manufacturing paving blocks by 5%). Studies have also focused on the use of paint sludge in construction materials as a substitute for aggregates, including cement and lime. The products obtained are lightweight building materials that provide insulation against heat and sound [23].

There is a need for further studies to assess the toxicity and potential environmental risks of the effluents generated, as well as different treatments that achieve the efficient removal of cosmetic pollutants [21]. The development of sustainable technologies for the production process and new ways of reusing industrial waste promote new ecologically-sound possibilities to overcome the limitations related to the scarcity and depletion of natural resources [24]. In addition to adding value to waste products that would previously have been discarded, new manufacturing industries may emerge from the use of the wastes, which is economically attractive.

### 3.2. Waste Generated during and after Consumption of Cosmetics

In addition to the generation of waste in the production processes, there is considerable concern regarding those that originate during and after the use of cosmetics, especially with regard to plastics. In the last years there has been a marked growth in plastic consumption, with an average annual increase of 2.5%. When it comes to quantity, approximately 355 million tons of consumption are generated annually [25]. Although PlaticsEurope aims for the reuse/recycling of at least 60% of all plastic packaging in the European Union by 2030, the relentless search for substitutes or improvements for petroleum-derived polymers continues [26].

Biodegradability is one of the factors that demonstrate when a product or material is environmentally friendly. Materials with low biodegradability can cause serious environmental damage if improperly discarded. Data show that from 1950 to 2015, approximately 8300 megatons of synthetic polymers were produced and, of these, 4900 megatons were discarded in landfills or in the environment [27]. Through the life cycle analysis (LCA) of a product/material, it is possible to determine the environmental impact from the acquisition of the raw material, through production, distribution and use, to disposal. However, LCA does not take into account economic, ethical and social aspects. LCA generates information to be used in decision-making related to product development and ecodesign, improvements to the production system and product choice at the consumer level. Biopolymers have potential for application in the food, chemical, nanotechnology, biotechnology, medicine and textile industries [28,29].

The best-known polymers are plastics and they are now considered as an urgent environmental problem. In 2015, approximately 4.9 billion metric tons were produced worldwide. The main types of plastics produced are derived from polyethylene (PE), polypropylene (PP), polystyrene (PS), expanded polystyrene (EPM) and urethane polyester (PEUR). PE is the plastic most commonly used in the production of cosmetics and food packaging. In addition, approximately 50% of plastics are associated with aggressive chemical monomers and by-products that can cause irreversible environmental consequences. Part of the plastic material, in the course of its degradation, becomes microparticles, known as microplastics (MPs), which further highlights the need for the appropriate management of plastic [29].

It is estimated that there are approximately 5.25 trillion plastic particles in the oceans, which together add up to almost 269 thousand tons. Approximately 280 million tons of plastic materials are produced worldwide each year, which are often not disposed of correctly and remain in the oceans, soil, landfills and dumps [30].

Microplastics act as an adsorbent and can be considered as carriers for various harmful contaminants, such as heavy metals (Al, Cd, Co, Cr, Cu, Hg, Mn and Pb), polycyclic aromatic hydrocarbons (PAHs), polychlorinated biphenyls (PCBs), pesticides and persistent organic pollutants (POPs). Therefore, these components in combination with MPs can lead to bioaccumulation and biomagnification in the food chain, with numerous harmful effects on marine biota, including the alteration of metabolic and reproductive activity, oxidative stress and cellular or subcellular toxicity [31,32].

Depending on their size, small plastic particles can be divided into microplastics (5 mm at 0.1 μm) and nanoplastics (<0.1 μm). Particles below 130 µm can accumulate in human tissue and, consequently, can release the previously mentioned toxins, additives and monomers, with carcinogenic activity, in addition to being harmful to the digestive system, since the human body is not able to digest plastics. Therefore, the generation of microplastics affects all ecosystems, in an irregular and uncontrolled way, leading to an unsustainable cycle [31]. Therefore, based on these factors, there is clearly a need for the aggregation of actions, changes and optimization in the production processes and in the products produced by the cosmetic industry.

## 4. Sustainable Innovations in the Cosmetic Industry

The cosmetics industry not only faces problems associated with the generation of waste microplastics directly from cosmetics or their packaging, together with the use of petroleum products but also issues related to animal testing, advances in the development of 3D skin models along with the emergence of organ-on-chip technology are reviewed and commented. Sun et al. shows the applications of skin-on-a-chip SOC along with 3D bioprinting technology as promising to build fully functional 3D skin products in the pharmaceutical and cosmetic industries [33].

Thus, a market has emerged focused on the creation and production of sustainable cosmetics, also referred to as ecological, green and natura products. It can be considered that ecological cosmetics take care of the skin using natural ingredients, such as herbs and other plants and essential oils, which are combined with carriers, preservatives, emulsifiers and natural humectants. In addition, companies commonly focus on the tripod of sustainability, by considering the environmental, social and economic impacts throughout the life cycle of the cosmetic product [34].

### 4.1. Sustainable Packaging Derived from Biopolymers

As previously reported, the most commonly used polymer in cosmetic products, in packaging and in the product itself, are plastics, more specifically, polyethylene. In view of the above-mentioned problems and harmful environmental impacts, the development of biopolymers as a new market has emerged and this area has been gaining strength in recent years. The market for biopolymers, which are rigid and flexible, is maintained by the growing demand in the packaging industry, in response to environmental legislation and government policies. The size of the biopolymers market was $6.95 billion in 2018 and is estimated to reach $14.92 billion by 2023, at a compound annual growth rate of 16.5% [35].

Biopolymers include plant derivatives, biomass, cellulose and even micro-organisms, many of which are noted for their excellent properties. Poly(lactic acid) (PLA) is a kind of aliphatic polyester, produced through the fermentation of sugar, where lactic acid (LA) is converted into lactide and eventually into PLA. It presents biodegradability, biocompatibility, non-toxicity, high mechanical resistance and a good cost-benefit ratio. PLA has been widely studied and used for food packaging, tissue engineering, cosmetics and drug distribution applications [36]. In particular, PLA is known as a polymer that degrades in sunlight, and there are records of complete degradation within 6 months to 1 year. In addition, the PLA production and disposal processes, compared to plastics derived from petrochemicals, contribute to reducing greenhouse gas (GHG) emissions [36]. A disadvantage is the low resistance to temperature (PLA softens at 60 °C), restricting its applicability, but copolymerization with heat-resistant polymers can help to address this problem [37].

Another widespread polymer is polybutylene succinate (PBS), which is obtained from the condensation polymerization of succinic acid (SA) and butanediol. Commonly, SA is the final product of the metabolization of anaerobic, aerobic or facultative micro-organisms. It is noted for its thermal and mechanical properties [38]. However, as with other biopolymers, analysis shows that the process of obtaining succinic acid by the biochemical route and polymerization is not yet an economically viable or competitive process in relation to the petrochemical route, given that petroleum is cheaper to produce [39].

Starch is synthesized from plants and is the second most abundant biodegradable polymer in the world. It accumulates as granules in plant cells and these granules have crystalline and amorphous regions. They are composed of amylose and amylopectin, and higher levels of amylose are desirable as this compound contributes to the film resistance, in addition to reducing the water solubility [40]. Commercially available starch is obtained mainly from potatoes and other sources include corn, rice and wheat. Starch-based polymers have high biocompatibility and low toxicity and they are biodegradable, with excellent mechanical properties. In addition, starch can be mixed with plasticizers, such as citric acid, sorbitol and glycerol, to further improve its properties and applicability [37,41].

The use of feedstock for products, such as biopolymers and biofuels can be an issue [42,43]. Land use for the production of raw material for biopolymers, such as corn zein, soy protein, starch, and wheat gluten [44], can compete for use in the food industry, generating a consequence of the ambitions of agricultural producers to participate as a supplier of raw material for biofuel companies and biopolymers [45]. Due to the development of biotechnology, nanotechnology and materials science, many sustainable biomaterials derived from natural resources, including carbonaceous materials, have been the subject of several studies [44,46,47] in the food industry, such as those from the processing of seafood, shrimp, shells and fruits, agricultural processes, and plastic waste recovered from the ocean. The development of biocomposites derived from multiple natural fibers, such as coconut and jute, has also been investigated [46].

Polyhydroxyalkanoates (PHA) show good potential to replace petroleum-derived plastics. PHAs are polyesters synthesized intracellularly by some micro-organisms, such as carbon, energy and fatty acids reserves, metabolized under stress conditions. These polyesters are biodegradable and originate from renewable carbon sources [39,40]. With regard to large-scale production, PHAs are often overlooked, given the high cost of acquiring expensive fermentation carbon sources, which account for approximately 40% of total PHA production costs [47,48]. In addition, it is relatively difficult to remove these polyesters from the micro-organisms [48].

A study by Uslu et al. (2019) was aimed at verifying the possibility of obtaining a better environmental profile for a cosmetic tube by applying life cycle analysis (LCA) and ecological design strategies. Three different cosmetic packages were investigated. The first consisted of high-density polyethylene (HDPE) + linear low-density polyethylene (LLDPE) + ethylene vinyl alcohol (EVOH) + adhesive. The second was made of Granic 742 + LLDPE + EVOH + adhesive and the third consisted of Granic 742 + -HDPE recycled post-consumer + EVOH + adhesive. To carry out environmental impact estimates with LCA, the researchers analyzed each of the packages by considering acidification, climate change, freshwater and marine eutrophication, photochemical ozone formation, sewage and water resources, depletion of minerals, and fossil and renewable energy resources, including primary energy from non-renewable sources and primary energy from renewable sources. The results of the study showed that cosmetic tubes with less environmental emissions can be obtained by changing virgin petro-chemicals by these materials at some portions, while maintaining its technical feasibility and reducing costs [49].

Based on the reported alternatives, several biotechnological processes have been proposed to produce biodegradable polymers, but most of them are not economically viable, especially for large-scale production, when compared with low cost conventional plastics obtained from fossil raw materials. Consequently, compared to the production of conventional plastics, which amounts to approximately 335 million tons/year, the mass production of biodegradable polymers is less than 1% [37].

### 4.2. Natural Preservatives

With regard to natural cosmetics, one of the current issues is the conservation of ingredients in a natural way, since preservatives are commonly made with petroleum derivatives. Research has shown some plants and derivatives that have antibacterial and antifungal action ensure greater durability and conservation of products. Notable examples are thyme, turmeric, garlic, tea tree and rosemary, which contain metabolites, such as lectins, alkaloids, terpenoids, polypeptides, polyphenols and polyacetylenes, that have antifungal and antibacterial action [50].

#### 4.2.1. Essential Oils

Essential oils (EOs) are products of the secondary metabolism of plants. They are involved in the pollination process and are produced in the organs in a natural way as a form of defense against animals and phytopathogens and in adaptation to sudden changes. There are several extraction methods available, including the use of steam dragging, cold pressing, supercritical fluids, enfleurage and solvents. Natural antioxidant, antimicrobial and anti-inflammatory agents are composed of chemical classes naturally produced by plants, and the classes present in EOs include monoterpenes, diterpenes, sesquiterpenes, phenylpropanoids and phenolic compounds [51].

Consequently, given these properties, EOs have become attractive for application in natural cosmetics. They are excellent candidates for the substitution of preservatives and antioxidants derived from petroleum, in addition to adding fragrance, allowing greater collagen production and acting in the treatment of skin dyschromias, among other benefits. The results of studies in this area could promote the discovery of efficient and stable natural antifungal condoms based on the nanoemulsion technique [50].

Although essential oils have excellent antifungal and antibacterial action, the complete removal of petrochemical preservatives from cosmetics and their replacement by substances of natural origin is not easy to achieve. The durability of antimicrobial action of EO is relatively low (2–3 months) compared to petrochemical preservatives, such as parabens. Extending the durability of EO action presents a significant challenge in the cosmetic industry. In this regard, studies on the nanoencapsulation of thymol have demonstrated an increase in the durability of the antimicrobial action of EOs. However, for application on a large scale, the global costs of materials and manufacturing exceed those associated with traditional and well-known methods of preservation [50].

#### 4.2.2. Microalgae

A line of research also focused on the development of sustainable cosmetics has emerged in recent years, that is, making use of the properties of microalgae in formulations, mainly as excipients, dyes, emulsion stabilizers, foams and thickeners (Bertsch et al., 2021). Extracts of the *Arthrospira* and *Chlorella* species can be found in refreshing and regenerating products, emollients, sun protection products, peels, creams to stimulate collagen synthesis, anti-aging products and even dyes, due to the presence of chlorophyll, carotenoids and phycobilins in microalgae. Red macroalgae (for example, porphyra and palmaria) contain high levels of arginine, a precursor to urea, added in cosmetic formulas [52].

Considering the multiple potential uses of algae in cosmetics, this area merits further research aimed at improving the viability of their large-scale application to replace the use of petroleum and its derivatives.

#### 4.2.3. Bacterial Cellulose

Bacterial cellulose (BC) is a biodegradable, biocompatible polymer that is resistant to traction and has high crystallinity and wettability. Its application in several areas is growing due to its excellent properties. The structure of BC is similar to that of vegetable cellulose; however, it lacks some constituents, such as lignin. BC is obtained from micro-organisms, such as Gluconacetobacter, Acetobacter, Agrobacterium, Achromobacter, Aerobacter, Sarcina, Azobacter, Rhizobium, Pseudomonas, Salmonella and Alcaligenes, via processes of biosynthesis and carbon consumption. In biomedicine, BC has been applied to reconstitute burnt and cut skin, and to build artificial eardrums. It is also widely used in the development of food packaging [53].

Regarding cosmetics, bacterial cellulose has a wide array of potential applications. Examples include for the delivery of active agents to the skin and as an emulsion stabilizer, thickener and emulsifier of creams. It can be combined with several active compounds, such as hyaluronic, moisturizing and antioxidant agents. Despite its potential applications in cosmetics, as with other biological processes, it is expensive to apply on an industrial scale. Thus, further research needs to be conducted to optimize the synthesis of BC [54].

Environmentally-friendly products, including green cosmetics, need to be effective and designed for environmental safety at every stage of their development, and they must be produced with minimal damage to the environment. Therefore, compared to conventional cosmetics, ecologically sound products are more appropriate and aligned with sustainable development, contributing to the sustainable growth of businesses with green strategies.

## 5. The Sustainable Development Goals (SDGs) as Guidance for the Cosmetic Industry

**Figure 2:**
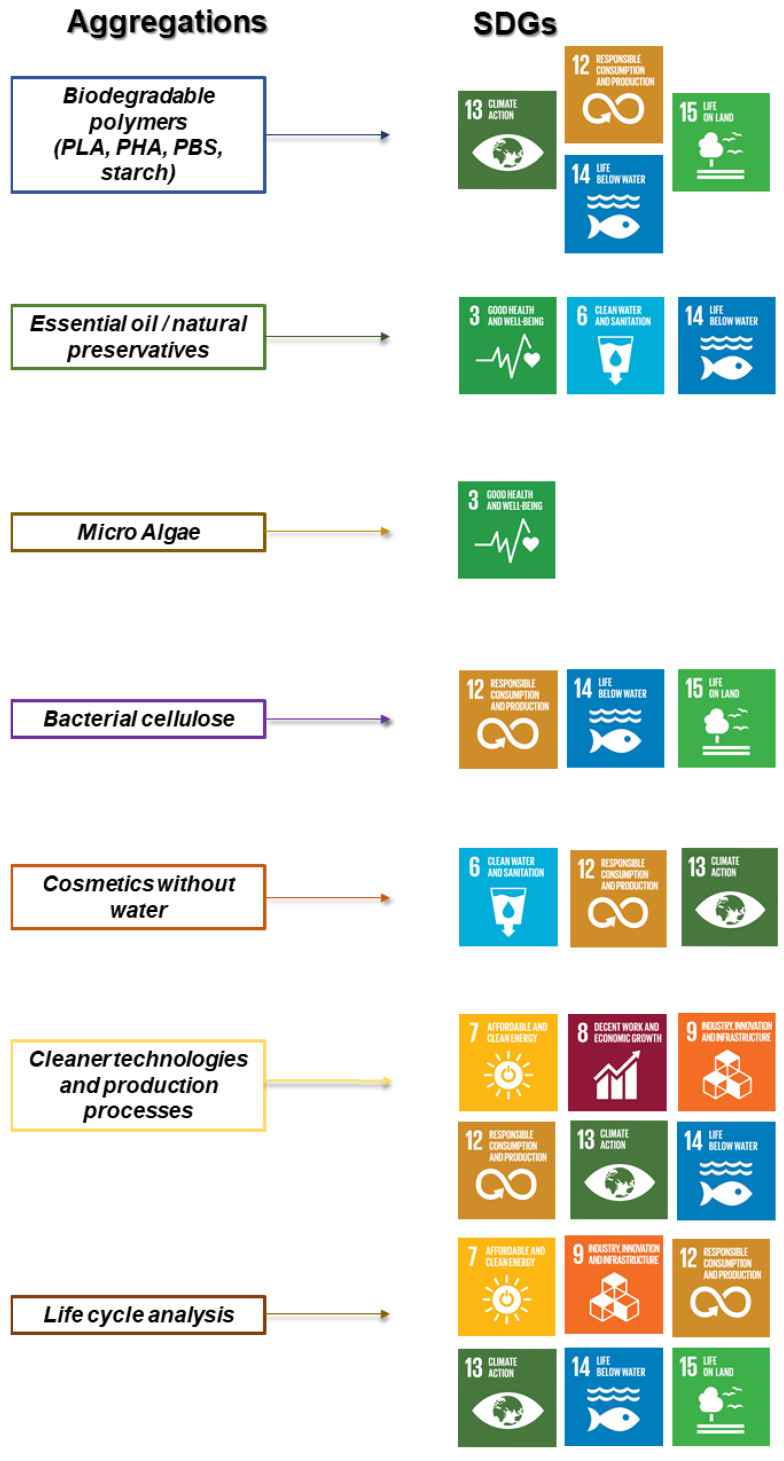
Highlights the SDGs that are most relevant to the cosmetic industry according to each aggregation of actions.

The 17 Sustainable Development Goals (SDGs) proposed by the UN are aimed at global well-being and social, environmental and economic sustainability [55]. Specifically, the SDGs 3, 6, 7, 8, 9, 12, 14, 13 deal with mitigating the impacts of water pollution, waste disposal and climate change. These objectives are relevant to the development of a more sustainable and environmentally-friendly cosmetics industry [56], the Figure 2 shows a relationship between the cosmetics industry aggregations and the SDGs.

The information detailed in this article provides an overview of the main issues currently confronting the cosmetics industry. These include pollution resulting from product components, especially when handled incorrectly in the extraction and production stages. These contaminants can reach aquatic environments, the air and a variety of ecosystems. Another issue is the wide use of petroleum derivatives, directly in cosmetics or in their packaging and, after acquisition by the consumer, it is difficult to ensure their correct disposal. The results of this are contaminated environments, compromised health and well-being, and insecurity regarding a more sustainable future. Aligning the practices of the cosmetics industry with the Sustainable Development Goals provides guidance on how to adapt in order to guarantee environmental sustainability along with socially safe and economically viable production processes.

It can be noted that the SDGs are strongly interconnected with each other, that is, one complements the other and vice versa. Thus, they should be perceived as forming a highly interconnected, interdependent and systemic network [57]. This perception is discussed in detail below based on the Figure 2.

Biopolymers as alternatives for cosmetic packaging are a possible solution for the accumulation of petroleum-derived plastics in nature, meeting the goals of SDGs 12, 13, 14 and 15. SDG 12 is related to the disposal of cosmetics packaging. Notably, target 12.5 aims, by 2030, to substantially reduce the generation of waste through prevention, reduction, recycling and reuse. Achieving this goal is complicated by the fact that the industry does not have control over the disposal of the packaging, as this is carried out by the consumer. It could be left for collection as ordinary domestic waste and later deposited in landfills or garbage dumps, or even be discarded irresponsibly in vacant lots, rivers and seas. At best, the consumer can perform the correct cleaning and separation of the plastic, which can then be recycled and transformed into a new product. Target 12.6 aims to encourage companies to adopt sustainable practices together with goal 12.4 that seeks to achieve environmentally sound handling of chemicals and all residues throughout the production process and significantly reduce their release to minimize their negative impacts.

SDGs 14 and 15 are focused on waste generation and the conservation of the marine environments and terrestrial biodiversity. Target 14.1 and 15.5 are aimed at the conservation and sustainable use of the oceans, seas and marine resources, and taking urgent and significant measures to reduce the degradation of natural habitats and halt the loss of biodiversity. Therefore, when there is unrestrained generation of waste (solid, liquid and gaseous) and there is no option for reuse or recycling, a suitable destination needs to be found to avoid the contamination of soil, air and water and the destruction of habitats by aggressive chemicals, surfactants, petrochemical derivatives, heavy metals and microplastics, commonly found in cosmetics [32,58].

Raw materials of natural origin, such as essential oil/natural preservatives, microalgae and bacterial cellulose, are a good option to mitigate the risk of using aggressive chemicals, meeting the goals of SDGs 3, 6, 12, 14 and 15. When considering health and well-being, the aggregation of natural substances, free of petroleum, parabens and microplastics, becomes more attractive and is aligned with the SDGs, especially SDG 3. This would demonstrate to the consumer that the industry is attentive to health aspects as well as all of the environmental issues previously mentioned in this article.

The cosmetic industry is strongly linked to SDG 3, as both are focused on health and human well-being, with a focus on hygiene, personal care and body maintenance [59]. However, aligned to the high production level of cosmetics, target 3.9 aims, by 2030, to substantially reduce the number of deaths and illnesses from hazardous chemicals and air, water and soil pollution and contamination. The production of cosmetics requires a large number of chemical agents, to ensure stable product formulations, with good odor, appearance and durability. Therefore, the management and disposal of wastewater from the production process must be carried out responsibly, taking care to avoid an irregular release of detergents, dyes and heavy metals into rivers, which can directly affect entire ecosystems.

New processes for producing cosmetics with little use of water have also emerged, innovations in the sector that meet the goals of the SDGs 6, 12 and 13. In the cosmetics industry, there is a need for substantial amounts of potable water with adequate quality parameters. Regarding the reuse of wastewater in this sector, there are few uses to which it can be applied, for instance, the cleaning of sidewalks and company sanitary facilities. Therefore, the search for methods to minimize wastage of potable water in the production processes could lead to responsible usage, for example, identifying possible leaks from valves and pipes, the installation of water meters to control consumption and avoiding the use of high quality water in applications where it is not required [13].

In this context, target 6.3 aims, by 2030, to improve water quality, reducing pollution substantially increasing recycling and safe reuse [55]. In the cosmetics industry, there is a need for substantial amounts of potable water with adequate quality parameters. Regarding the reuse of wastewater in this sector, there are a few uses to which it can be applied, for instance, the cleaning of sidewalks and company sanitary facilities. Therefore, the search for methods to minimize wastage of potable water in the production processes could lead to responsible usage, for example, identifying possible leaks from valves and pipes, the installation of water meters to control consumption and avoiding the use of high quality water in applications where it is not required [60]

The cosmetics industry has increasingly invested in clean technologies with new production processes, meeting SDGs 7, 8, 9, 12, 13 and 14. The SDG 9 “Industry, innovation and infrastructure” appears to be best positioned to link to the other objectives that involve the aggregation of actions to the cosmetic industry, given that target 9.4 appears as the basis for sustainable industrial modernization, with increased efficiency in the use of resources and adoption of clean and environmentally-friendly industrial technologies and processes. The cosmetics industry, is highly dependent on natural, fossil and other resources, and greater efficiency in their use is attractive in the long run from the economic and, mainly, environmental perspective. Aggregating clean and sustainable technologies is essential in the entire production process, based on technologies developed through innovation and R&D. It should be noted that innovation is the main impetus of many countries in the search for resilient and inclusive industrial development [57].

Regarding the use of renewable energy in the cosmetic industry, target 7.2 aims to increase the share of renewable energies in the global energy matrix. The production of cosmetics is an economic activity of great scope and proportions and, consequently, there are higher levels of energy demand. Therefore, the use of energy from renewable resources could reduce adverse environmental impacts by lowering the levels of air pollution and greenhouse gas (GHG) release [61,62]. In this context, target 13.3 seeks to raise awareness about global climate mitigation, adaptation, impact reduction, and early warning of climate change. Global warming leads to sea level rise, increases in terrestrial temperatures, disrupts the equilibrium of ecosystems and, consequently, can provoke environmental catastrophes [63,64].

Assessing the complete life cycle of cosmetics has also been a concern of the sector, meeting SDGs 7, 9, 12, 13, 14 and 15. An interesting practice that can be adopted in this regard is ecodesign. This is based on developing the product with ecologically-sound practices applied throughout the process. This starts with the choice of raw materials, considering the environmental impact and recyclability, assessing the ability to reuse the components and design them with an extended service life. In addition, the use of energy based on renewable resources is crucial and redesign needs to be allowed to take advantage of the main opportunities for changes in production processes. The priority in ecodesign is to eliminate procedures with a high toxicity index, aimed at achieving cleaner production processes and more competitive products [65].

When it comes to packaging, approximately 80% of the environmental impact is assessed in the design process. Ecodesign can include a strategy for the return of waste materials from customers to the company, often using the same system where waste is collected for reuse. Additionally, packages could be redesigned so that they contain derivatives of biodegradable raw material, are of lighter weight and make less use of labels. There are also reports of software developed for companies to facilitate packaging ecomodulation, which calculates the environmental impacts even before the packaging is development, and this approached has been used in Italy and Belgium [65,66].

Therefore, the change needed in the industrial sector, with regard to the generation of waste, is to evolve toward eco-innovation, aligning ecodesign and life cycle analysis. This could lead to a reduction in environmental impacts, through the development of new technologies and products, and the proposing of concepts of innovative, resilient production processes that guarantee improvements in favor of sustainability [54,55]. In this context, SDG 8 can also be mentioned, which seeks precisely to promote sustained, inclusive and sustainable economic growth [55].

Finally, target 12.8 seeks to ensure that people, everywhere, have relevant information and awareness for sustainable development and lifestyles in harmony with nature. If there is no reinforcement of consumer awareness, they will not be the commitment, collaboration and mobilization of industries, researchers and NGOs [67]. To enhance the awareness of the general population it is necessary to provide consumers with adequate information on sustainability at the product level, including appropriate disposal practices, with labeling indicating the correct way to dispose of the product for recycling, even showing collection points in the case of reuse. Several other initiatives are directed toward companies and consumers, aimed at generating awareness, promoting innovation and disseminating best practices [64].

## 6. Final Considerations and Future Perspectives

Achieving sustainable development is not solely dependent on a portion of the population. To ensure that “no one is left behind” there must be partnerships with all stakeholders, including political leaders and government departments, researchers and universities, NGOs, civil society, agents of the law and business people, all united and cooperating in order to fulfill the 2030 Agenda to guarantee the permanence of resources for future generations.

It is up to scientists and academics to conduct research and analysis, develop methodologies and perform tests so that, based on scientific data, actions for sustainable development are put into practice by civil society. In addition, governments are responsible for planning, supporting and assisting universities and researchers, with investments in scholarships, laboratories and technologies to allow research to be carried out.

It is also up to government officials, business leaders and law enforcement officials to prepare for and comply with regulations and laws, with fair and concrete punishments for industries, companies and citizens who commit environmental crimes, which also affect socioeconomic spheres. As an example, the French government proposed to end, by 1 January 2018, the commercialization of rinsed cosmetic products for exfoliation or cleaning, containing solid plastic particles with the exception of particles of natural origin. In June 2018, the Scottish government made it a crime to manufacture any personal hygiene product with a rinse using plastic microspheres. Sentencing involves a fine not exceeding £5000, for conviction or a prison term of no more than 2 years [68].

As a civil society, it is necessary to rethink our attitudes in relation to the use of plastics, the disposal of the residues we produce, the separation of waste according to the type for disposal (recycling or landfill), promoting sustainable behaviors by breaking old habits and adopting new ones. In addition, it is attractive to create open forums for communicating knowledge to citizens to promote an awareness of hazards associated with plastic waste and, in addition, television advertisements with a wide range of environmental impacts. We also need to rethink our consumption habits because in view of all the issues addressed herein, there is a need for long-term efforts to bring about behavioral changes, which could be lost due to interruption or conflicting information [69].

These aspects together demonstrate that achieving development based on sustainability is dependent on the joint action of each entity belonging to society in its designated function [70].

The cosmetics industry has great relevance in the global market, gaining millions of consumers year after year. As a result, it is important to study all the product production processes and to identify the socio-economic-environmental impacts generated throughout the life cycle, from the conception of raw materials to the final disposal carried out by the consumer. Seeking to align themselves with sustainable development, many industries are changing their outlook and looking for more ecologically acceptable alternatives for the development of their products.

The Sustainable Development Goals are an excellent guide for companies that want to adapt to technological innovations, making use of cleaner technologies, the aggregation of ecologically sound components in the product and its packaging, reducing the use of aggressive chemicals that directly adversely affect the environment and human health, and seeking to add value to the residues from the production stages and to the end use by the consumer. Companies that are already aligned with sustainability have stood out in the market, surpassing their competitors and gaining more consumers each year, given the increase in the environmental awareness of consumers, especially with regard to the ethical and the ecological principles of each industry.

Therefore, economic growth must be accompanied by technological changes, the efficient use of natural resources, the use of clean energy and biomaterials, such as biopolymers, aggregating ecological actions, and social responsibility, in order to mitigate the effects of the expansion of production on the environment. It is also expected that companies will lead this transformation, motivating the sustainability of the market, developing sustainable approaches, encouraging other companies to aggregate ecological actions, focusing on achieving sustainable development with resilient industries.

## Data Availability

Not applicable.
